# Communication strategies for rare cancers: a systematic review protocol

**DOI:** 10.1186/s13643-019-1017-5

**Published:** 2019-04-23

**Authors:** Catherine Bell, Katie Kerr, Kerry Moore, Charlene McShane, Lesley Anderson, Amy Jayne McKnight, Helen McAneney

**Affiliations:** Centre for Public Health, School of Medicine, Dentistry and Biomedical Sciences, Queen’s University Belfast, Belfast, UK

**Keywords:** Rare Cancer, Communication, Strategies, Information, Healthcare professional, Carers, Patients

## Abstract

**Background:**

Rare cancers comprise almost a quarter of all cancers in Europe, and patients generally have poorer outcomes than those suffering from more common cancers. This is attributed in part to a general lack of knowledge and awareness of rare cancers. This review aims to examine the communication strategies being used throughout the world to inform on rare cancers and to highlight any opportunities for improvement.

**Methods:**

A systematic review of literature published in English prior to November 2018 will be conducted, screening articles from the electronic databases MEDLINE, PubMed, EMBASE, Web of Science, PsycINFO, CINAHL Plus and the Cochrane Database of Systematic Reviews. Grey literature databases (GreyLit, OpenGrey) will also be searched in order to screen for any unpublished works. As well as primary literature, reference lists will be examined via forward and reverse citation screening. The review will be reported using the Preferred Reporting Items for Systematic Reviews and Meta-Analysis (PRISMA). Titles and abstracts will first be examined for eligibility, with remaining studies undergoing a full-text screening before being included in the final review. Individual studies will be screened for bias, and a meta-analysis performed provided there is enough data. If insufficient homogenous literature exists, a narrative summary of the literature will be produced.

**Discussion:**

Despite the broad topic and width of study type that will be considered, this review hopes to provide a reflective summary of the communication strategies available for people living with and working with rare cancer. It aims to reveal any gaps in the resources available, to contribute to the long-term improvement of diagnosis and management of rare cancers.

**Systematic review registration:**

PROSPERO CRD42018099784

**Electronic supplementary material:**

The online version of this article (10.1186/s13643-019-1017-5) contains supplementary material, which is available to authorized users.

## Background

The definition of rare cancer varies throughout the world. In Europe, a cancer is described as “rare” if it has an incidence of less than 6 per 100,000 diagnosed people per year [1], whilst in the USA, the threshold is 15 new diagnoses per 100,000 per year [2]. Consequently, some cancers which are considered “rare” in one country/region may be considered “common” in another. For example, under the European Union, Surveillance of Rare Cancer in Europe (RARECARE) definition, cancers such as adenocarcinoma (with variants) of the colon/rectum/pancreas/lung/corpus uteri/ovary/prostate, renal cell carcinoma (with variants) and haematological malignancies such as multiple myeloma/plasmacytoma (and heavy chain disease) and diffuse B cell lymphoma are classified as rare in Europe but are considered common in the USA [3, 4].

Despite the name, rare cancers collectively account for around 24% of all cancer diagnoses in Europe [3], and 20% of those in the USA [4]. One reason why the frequency of cancer diagnoses is thought to be increasing is due to the introduction of molecular techniques, combining with previously used histological techniques, to diagnose and classify cancer types. Such techniques have caused cancer types that were previously classified as “common” to instead be seen as a larger group of molecularly distinct rare cancers [5].

Although rare cancers as a whole constitute a significant health concern to the general public, the scarcity of patients with an individual rare cancer type within defined geographic areas makes it very difficult to produce clinical trial data that is statistically reliable [6]. Patients suffering from rare cancers experience significantly poorer outcomes than patients suffering from more common cancers, with the average 5-year survival for rare cancers being up to 20% lower than that of a common cancer [4]. International collaborations have proved beneficial, particularly for the research of extremely rare cancers, but funding is difficult to obtain and little work has been done to identify treatment options or to improve understanding of rare cancer development [6].

Another contributor to the poorer outcomes in patients with rare cancers is the time to diagnosis. Patients with rare cancers tend to be diagnosed at a later stage and are often initially misdiagnosed with a more common cancer [7]. Delayed diagnosis is detrimental to patient prognosis. Alongside a shortage of sufficient specialised facilities, this delay in diagnosis is partly due to a lack of awareness and knowledge of rare cancers by health professionals [8] and patients alike, which reflects a wider gap in the understanding of these diseases throughout the general population. This has a negative impact on communication and understanding for all affected by rare cancers. For example, a study carried out in England reported 43.3% of myeloma patients waited more than 3 months to present to their general practitioner. A third of all patients included in the study (including myeloma, leukaemia and lymphoma patients) said they were not aware that their symptoms were serious [9]. Social media has revolutionised healthcare, allowing efficient communication and exchange of information between health professionals and the general public [10]. However, the implications for communication surrounding rare cancers have not been examined.

### Review aim and objectives

This review aims to examine the current literature surrounding communication of information on rare cancers by:

1. Comprehensively identifying and evaluating publications pertaining to strategies for rare cancer information communication
2. Providing a synthesis of the types of strategies available and their global accessibility
3. Highlighting who these strategies are targeted for (patients, carers, healthcare professionals, the general public) and the perceptions/understanding of the communication intervention by these groups where available.

### Rationale for review

Rare cancers collectively account for more cases of cancer than any individual type [11] and therefore represent an important health problem. A general lack of knowledge contributes to the high proportion of misdiagnosis, amongst other factors [12]. This review aims to shed light on the communication strategies available to aid understanding of rare cancers, with the hope of highlighting possible avenues for improvement in the information available. In the long term, this could lead to a general increase in the awareness and knowledge of rare cancers and hopefully improve on early diagnosis and minimise misdiagnoses by shortening the diagnostic pathway. Increased communication and understanding could also improve management by carers and healthcare professionals, thereby improving outcomes, such as long-term survival, for those affected by rare cancer.

## Methods

This protocol was created using guidance stated on the BMC website [13] and using the Preferred Reporting Items for Systematic Review and Meta-Analysis Protocols (PRISMA-P) checklist (see Additional file 1). This protocol has been registered on the PROSPERO database of prospectively registered systematic reviews (Reference: CRD42018099784).

### Inclusion criteria

The inclusion criteria were designed in reference to the Population, Intervention, Comparison, Outcome (PICO) framework [14], although certain aspects of this framework are not completely applicable to the topic of the review.

#### Study population

Only studies involving human participants will be considered. There is no age restriction for human participants.

#### Intervention

The interventions highlighted in this review will include a range of communication strategies used to convey information about rare cancers (for example treatment, diagnosis and general awareness). The rare cancer described in each study must meet the criteria for a rare disease in their place of research, e.g. the European or American definition of a rare cancer.

#### Comparator

Where applicable, this review will compare studies of rare cancer within a single group of participants, pre- and post-implementation of a communication intervention strategy. Studies which meet the population, intervention and outcome elements of our eligibility criteria but not this comparator will still be included.

#### Outcome(s)

Data on the range of communication strategies that have been used when informing on rare cancers will be examined.

By examining the origin of publication, this review will also examine the accessibility of different communication strategies that are available globally.

If available, information on how well the intended audience understood the information provided using the communication strategies will be considered; however, due to the limited literature in the area, it is predicted that information regarding patient understanding may not be available.

#### Types of study to be included

There is no restriction on the type of study eligible for this review.

#### Time frame

All literature published in November 2018 and prior are eligible. There is no restriction on the time frame of studies that will be considered, to encapsulate as many relevant studies as possible.

#### Context

Publications from any country of origin are eligible. Publications must be written in English.

### Information sources

Several bibliographic databases will be searched for relevant material for inclusion in the review. Such databases include MEDLINE, PubMed, EMBASE, Web of Science, PsycINFO, CINAHL and the Cochrane Database of Systematic Reviews. Grey Literature databases GreyLit and OpenGrey will also be searched. As well as screening primary publications, forward and reverse citation screening will also be undertaken.

A search strategy will be created and adapted to each database. Search terms will be developed from several sources, including ‘RareCareNet’ [15], ‘Cancer Caring Coping’ [16], ‘Rare Cancers Europe’ [17] and similar organisations worldwide. The terms will include keywords relevant to communication and delivery of information, different forms of communication strategies and phrases related to the intended audience, whether it be patients, carers or healthcare professionals.

### Search strategy

A draft of the search strategies to be used for MEDLINE and other databases is attached (see Additional file 2).

### Study records

#### Data management

Data will be managed using online reference management tools such as EndNote. These will also be used to record reasons for exclusion in each case.

#### Selection and data collection process

Initial search results from all databases will be screened for duplicates. Titles and abstracts will then be screened and excluded if they are not considered relevant to the review topic. Finally, full text will be screened to confirm eligibility of the publication. A summary of the selection process will be made into a flow diagram following the PRISMA design [18].

Two independent reviewers (CB and KK) will conduct the search, examine data and ensure papers meet the inclusion criteria mentioned previously, with any disagreements to be resolved by discussion. If a consensus cannot be reached, a third independent individual (KM) will make the final decision. Data will be extracted, in duplicate, by two or more independent reviewers and collected into a table such as that of Table 1. This table will be checked and should there be any disagreements, these will be discussed with a third co-author (HMcA) who will make the decision if consensus cannot be reached. If additional or further information other than that contained in the publication is required, attempts to contact the original authors will be made.

**Table 1:** Descriptors of data to be extracted from included studies

Author	Year of publication	Country	Study design	Cancer type investigated (if applicable)	Communication strategy	Intent of communication strategy	Intended audience	Population size (if applicable)	Findings	
									Quantitative	Qualitative
Paper A authors	xxxx	UK	[Type]	[Rare cancer type]	Leaflet in a General Practice reception	General Information to increase awareness about [rare cancer type]	Patient, public	X	X number of people said they would pick up the leaflet, Y number said they found the information useful	There was an increase in undiagnosed patients raising concerns about [rare cancer]; however, the content of leaflet was no more informative than that provided by online
Paper B authors	xxxx	USA	[Type]	[Rare cancer type]	Online discussion forum	Discussion between patients suffering from [a rare cancer] and experts in the field	Patient, healthcare professionals, research specialists	X	X people viewed the forum, Y people commented, X% were patients and X% were healthcare professionals	Helped understanding, allowed communication between affected individuals
Paper C authors	xxxx	Australia	[Type]	[Rare cancer type]	Text	Test result of a novel pharmaceutical agent	Patient	X	X number of people were involved in the study	Patients understood implications of the result

An example table of data that will be extracted from each study. If several studies examine homogenous interventions, these results will be included and a meta-analysis will be performed

#### Quality assessment

Studies will be critically appraised to assess methodological quality using the appropriate checklists for the study design, available from the Joanna Briggs Institute [19] and the Critical Appraisal Skills Programme (CASP) [20]. As these tools do not provide a quantitative score, studies will be assigned as having either weak, moderate or strong methodological rigour.

### Data synthesis

If there are three or more publications investigating the same area with the same outcomes, a meta-analysis shall be performed. However, due to the scarce nature of literature surrounding rare cancer, as well as the wide-ranging nature of communication strategies, it is expected that there will be insufficient data available for a meta-analysis.

Dependent on the number of studies to meet the inclusion criteria, a narrative synthesis or, more likely, a descriptive summary will be the most suitable way to present the findings from this review [21]. However, should the bodies of literature be heterogeneous, where the same problem has been conceptualised and investigated in different ways, then a meta-narrative approach to the synthesis will be contemplated [22, 23]. As such, each publication will be examined and information such as the following items extracted and considered:

i) Type of communication strategy employed
ii) Response to strategy
iii) Place of publication
iv) Date of publication

By doing this, any particularly widely used communication strategy will be identified, as well as any shortcomings in data availability. These will identify any discrepancies in accessibility to communication strategies and information. It will also be possible to observe if there is particular focus on certain rare cancer types over others. By looking at the date of publication, it will be possible to observe whether the number of studies on communication in rare cancer is increasing over time, as more and more rare cancers are discovered.

## Discussion

A significant proportion of all cancers are considered rare cancers. Despite this, there has been comparatively little research into the area. This review aims to look at the communication strategies in place for providing information about rare cancer, particularly as the importance of the internet and social media in healthcare become more apparent. For instance, the work by Pemmaraju and colleagues considers the creation and use of disease-specific hashtags on Twitter, for example #MPNSM (myeloproliferative neoplasms on social media), for the dissemination of information and facilitated interaction of healthcare stakeholders all over the world in the field of myeloproliferative neoplasms [24]. Other aspects of communication are reflected in the work of Peate which emphases the importance of effective health education in the case of testicular cancer, which although a rare cancer is the commonest malignancy in the UK among men aged 20–34 years and is the most curable [25]. The benefit of consortiums and collaborations in diagnosis and survivorship of rare cancers is considered by Blay and colleagues [26]. Additionally, there is the benefit of the provision of information to patients with rare cancers such that they can make informed decisions, as reported by Schultz, in the case of addressing the needs of patients with medullary thyroid carcinoma through the use of internet discussion forums [27]. These are just some of the communications strategies envisaged to be robustly reported on from the completed protocol.

Due to the scarcity of rare cancer literature as a whole, it is expected that there will be relatively few publications examining such communication strategies directly. Therefore, this review has designed a thorough search strategy that will encapsulate as many relevant publications as possible, such as additional inclusion of studies in which communication and rare cancer is not the primary focus. For example, we would include a study that examined how patients were informed as part of a larger investigation looking at a specific intervention for a specific rare cancer type, as well as studies which focus on communication and rare cancer only.

This broad approach aims to ensure that the resulting review is able to compare several studies and encapsulate all the ways in which information about rare cancer is communicated. As such, the studies included are unlikely to have homogenous interventions and outcomes, meaning a narrative synthesis of the data extracted will likely be more feasible than the preferable meta-analysis.

In order to identify English-based communication resources that can be easily adapted to the UK setting, only studies published in English will be eligible for inclusion. Whilst it is expected that some important non-English-language studies may be missed by this approach, a comprehensive search strategy that includes grey literature searches will be employed to ensure that all relevant English language studies are identified.

Despite the challenges this review faces in analysing literature in a developing field of research, the data discovered will be valuable in identifying areas of communication in rare cancer which need improvement. This information could inform healthcare professionals, carers and patients alike, leading to a raised awareness which could have many beneficial outcomes, including shortening the diagnostic pathway and improving the prognosis for those suffering from a rare cancer [28].

## Additional files

Additional file 1: PRISMA-P Checklist.pdf- A copy of the PRISMA-P checklist, including page number for each applicable sections stated in the checklist. (PDF 93 kb)

Additional file 2: Search Strategies.pdf. An example of the search terms to be used for MEDLINE and other databases. Similar terms will be used across all databases. (PDF 83 kb)

## Abbreviations

CASP: Critical Appraisal Skills Programme
PICO: Population, Intervention, Comparison, Outcome
PRISMA: Preferred Reporting Items for Systematic Review and Meta-Analysis

## References

[1] Gatta G, van der Zwan JM, Casali PG, Siesling S, Dei Tos AP, Kunkler I (2011). RARECARE working group. Rare cancers are not so rare: the rare cancer burden in Europe. Eur J Cancer.

[2] Greenlee RT, Goodman MT, Lynch CF, Platz CE, Havener LA, Howe HL (2010). The occurrence of rare cancers in US adults, 1995-2004. Public Health Rep.

[3] Gatta G, Capocaccia R, Botta L, Mallone S, De Angelis R, Ardanaz E (2017). Burden and centralised treatment in Europe of rare tumours: results of RARECAREnet—a population based study. Lancet Oncol.

[4] DeSantis CE, Kramer JL, Jemal A (2017). The burden of rare cancers in the United States. CA Cancer J Clin.

[5] Boyd N, Dancey JE, Gilks CB, Huntsman DG (2016). Rare cancers: a sea of opportunity. Lancet Oncol..

[6] Blay JY, Coindre JM, Ducimetière F, Ray-Coquard I (2016). Rare cancers: the value of research collaborations and consortia in rare cancers. Lancet Oncol.

[7] Very rare cancers- a problem neglected (2001). Lancet Oncol.

[8] Van der Graff W. Why rare cancers deserve a greater focus and a more targeted approach. Instit Cancer Res. 2018; https://www.icr.ac.uk/blogs/science-talk-the-icr-blog/page-details/why-rare-cancers-deserve-a-greater-focus-and-a-more-targeted-approach. Accessed 12 Jun 2018.

[9] Howell DA, Warburton F, Ramirez AJ, Roman E, Smith AG, Forbes LJL (2015). Risk factors and time to symptomatic presentation in leukaemia, lymphoma and myeloma. Br J Cancer.

[10] Zhou L, Zhang D, Yang CC, Wang Y (2018). Harnessing social media for health information management. Electron Commer Res Appl.

[11] Gatta G, Capocaccia R, Trama A, Martínez-García C, RARECARE Working Group (2010). The burden of rare cancers in Europe. Adv Exp Med Biol.

[12] Trama A, Marcos-Gragera R, Pérez MJS, van der Zwan JM, Ardanaz E, Bouchardy C (2016). Data quality in rare cancers registration: the report of the RARECARE data quality study. Tumori J.

[13] BMC Systematic Reviews: Protocol. https://systematicreviewsjournal.biomedcentral.com/submission-guidelines/preparing-your-manuscript/protocol (2016). Accessed 11 Jun 2018.

[14] Richardson WS, Wilson MC, Nishikawa J, Hayward RSA (1995). The well-built clinical question: a key to evidence-based decisions. ACP J Club.

[15] RARECARENet. Information network on rare cancers. http://www.rarecarenet.eu/ (2008). Accessed 18 Jun 2018.

[16] Santin O, McShane T, Fowler C, Mc Manus M, Mills M, Morrison J (2018). Cancer caring coping: the co-design development and evaluation of an online resource to support cancer caregivers. A report for HSC R&D Office.

[17] Rare Cancers Europe. http://www.rarecancerseurope.org/ (2018). Accessed 18 Jun 2018.

[18] Moher D., Liberati A., Tetzlaff J., Altman D. G (2009). Preferred reporting items for systematic reviews and meta-analyses: the PRISMA statement. BMJ.

[19] University of Adelaide (2017). The Joanna Briggs Institute.

[20] Critical Appraisal Skills Programme (2018). CASP Checklists.

[21] Popay J, Roberts H, Sowden A, Petticrew M, Arai L, Rodgers M (2006). Guidance on the conduct of narrative synthesis in systematic reviews: a product from the ESRC Methods Programme.

[22] Greenhalgh T, Robert G, Macfarlane F, Bate P, Kyriakidou O, Peacock R (2005). Storylines of research in diffusion of innovation: a meta-narrative approach to systematic review. Soc Sci Med (1982).

[23] Wong G, Greenhalgh T, Westhorp G, Buckingham J, Pawson R (2013). RAMESES publication standards: meta-narrative reviews. BMC Med.

[24] Pemmaraju N, Utengen A, Gupta V, Kiladjian JJ, Mesa R, Thompson MA (2017). Rare cancers and social media: analysis of Twitter metrics in the first 2 years of a rare-disease community for myeloproliferative neoplasms on social media-#MPNSM. Curr Hematol Malig Rep.

[25] Peate I (1997). Testicular cancer: the importance of effective health education. Br J Nurs.

[26] Blay JY, Jm C, Ducimetiere F, Ray-Coquard I (2016). The value of research collaborations and ocnsrtia in rare cancers. Lancet Oncol.

[27] Schultz PN (2002). Providing information to patients with a rare cancer: using Internet discussion forums to address the needs of patients with medullary thyroid carcinoma. Clin J Oncol Nurs.

[28] Neal RD, Tharmanathan P, France B, Din NU, Cotton S, Falon-Ferguson J (2015). Is increased time to diagnosis and treatment in symptomatic cancer associated with poorer outcomes? Systematic review. Br J Cancer.

