# Supplementary material for: Communication strategies for rare cancers: a systematic review protocol
Source: Syst Rev. 2019 Apr 23;8:102. doi: 10.1186/s13643-019-1017-5 (PMC6477728; doi:10.1186/s13643-019-1017-5)
Supplement: Supplementary file 2 — Search Strategies.pdf. An example of the search terms to be used for MEDLINE and other databases. Similar terms will be used across all databases. (PDF 83 kb) [file 13643_2019_1017_MOESM2_ESM.pdf]

Communication Strategies in Rare Cancers: A Systematic Review Protocol.

Catherine Bell, Katie Kerr, Kerry Moore, Charlene McShane, Lesley Anderson, Amy Jayne McKnight, Helen McAneney.

Additional File 2. Search strategy in various databases, being adapted for each database as necessary.

Search Strategy in MEDLINE

#	Search Term
1.	(rare adj5 (cancer* or carc* or neoplas* or malignan* or adenocarcinoma* or sarcoma* or tumo?r* or carc*)).mp.
2.	communication/ or "cell phone use"/ or information seeking behavior/ or literacy/
3.	writing/ or medical writing/ or research report/
4.	((oral* or verbal* or spoken) adj5 communicat*).mp.
5.	Medical Illustration/
6.	health education/ or consumer health information/ or health literacy/ or health fairs/ or health promotion/ or patient education as topic/
7.	mass media/ or motion pictures/ or radio/ or television/ or videodisc recording/ or compact disks/ or cd-i/ or cd-rom/ or videotape recording/
8.	Media ADJ0 campaign*.mp.
9.	Internet/ or blogging/ or social media/
10.	(twitter* or tweet* or facebook* or wordpress* or "social network*" or instagram* or snapchat*).mp.
11.	(leaflet* or pamphlet* or flyer* or brochure*).mp.
12.	webcasts/

13.	podcast*.mp.
14.	education, medical/ or education, medical, continuing/ or education, medical, graduate/ or "internship and residency"/ or education, medical, undergraduate/ or teaching rounds/
15.	Caregivers/
16.	education, dental/ or education, dental, continuing/ or education, dental, graduate/ or education, nursing/ or education, nursing, associate/ or education, nursing, baccalaureate/ or education, nursing, continuing/ or education, nursing, diploma programs/ or education, nursing, graduate/ or nursing education research/ or education, pharmacy/ or education, pharmacy, continuing/ or education, pharmacy, graduate/ or pharmacy residencies/ or education, public health professional/
17.	dentist-patient relations/ or nurse-patient relations/ or physician-patient relations/ or researcher-subject relations/
18.	"breaking bad news".mp.
19.	doctor ADJ0 patient ADJ0 communication*.mp.
20.	internet ADJ0 forum*.mp.
21.	community networks/ or social support/ or psychosocial support systems/
22.	((support* or survivor*) adj5 group*).mp.
23.	congresses as topic/ or consensus development conferences as topic/ or consensus development conferences, nih as topic/
24.	call centers/ or call centres/ or hotlines/
25.	help?line*.mp.
26.	2 or 3 or 4 or 5 or 6 or 7 or 8 or 9 or 10 or 11 or 12 or 13 or 14 or 15 or 16 or 17 or 18 or 19 or 20 or 21 or 22 or 23 or 24 or 25
27.	1 and 26

Web of Science Search Strategy

Search #	***Results***	***Search Terms***
# 26		#25 AND #1
# 25		#24 OR #23 OR #22 OR #21 OR #20 OR #19 OR #18 OR #17 OR #16 OR #15 OR #14 OR #13 OR #12 OR #11 OR #10 OR #9 OR #8 OR #7 OR #6 OR #5 OR #4 OR #3 OR #2
# 24		(TS=(help?line*))
# 23		(TS=(call centers/ or hotlines/))
# 22		(TS=((support* or survivor*) NEAR/5 group*))
# 21		(TS=(community networks/ or social support/ or psychosocial support systems/))
# 20		(TS=("internet forum*"))
# 19		(TS=("doctor NEAR/0 patient NEAR/0 communication*"))
# 18		(TS=("breaking bad news"))
# 17		(TS=(dentist-patient relations/ or nurse-patient relations/ or physician-patient relations/ or researcher-subject relations/))
# 16		(TS=(education, dental/ or education, dental, continuing/ or education, dental, graduate/ or education, nursing/ or education, nursing, associate/ or education, nursing, baccalaureate/ or education, nursing, continuing/ or education, nursing, diploma programs/ or education, nursing, graduate/ or nursing education research/ or education, pharmacy/ or education, pharmacy, continuing/ or education, pharmacy, graduate/ or pharmacy residencies/ or education, public health professional/))
# 15		(TS=(Caregivers/))
# 14		(TS=(education, medical/ or education, medical, continuing/ or education, medical, graduate/ or "internship and residency"/ or education, medical, undergraduate/ or teaching rounds/))
# 13		(TS=(podcast*))
# 12		(TS=(webcasts/))
# 11		(TS=(leaflet* or pamphlet* or flyer* or brochure*))
# 10		(TS=(twitter* or tweet* or facebook* or wordpress* or "social network*" or instagram* or snapchat*))
# 9		(TS=(Internet/ or blogging/ or social media/))

# 8		(TS=(Media NEAR/0 campaign*))
# 7		(TS=(mass media/ or motion pictures/ or radio/ or television/ or videodisc recording/ or compact disks/ or cd-i/ or cd-rom/ or videotape recording/))
# 6		(TS=(health education/ or consumer health information/ or health literacy/ or health fairs/ or health promotion/ or patient education as topic/))
# 5		(TS=(Medical Illustration/))
# 4		(TS=((oral* or verbal* or spoken) NEAR/5 communicat*))
# 3		(TS=(writing/ or medical writing/ or research report/))
# 2		(TS=(communication/ or "cell phone use"/ or information seeking behavior/ or literacy/))
# 1		TS=(rare near/5 (cancer* or carc* or neoplas* or malignan* or adenocarcinoma* or sarcoma* or tumo?r*))

CINAHL Plus Search Terms

Search Number	Search Term
S8 AND S62	
S62	S9 OR S10 OR S11 OR S12 OR S13 OR S14 OR S15 OR S16 OR S17 OR S18 OR S19 OR S20 OR S21 OR S22 OR S23 OR S24 OR S25 OR S26 OR S27 OR S28 OR S29 OR S30 OR S31 OR S32 OR S33 OR S34 OR S35 OR S36 OR S37 OR S38 OR S39 OR S40 OR S41 OR S42 OR S43 OR S44 OR S45 OR S46 OR S47 OR S48 OR S49 OR S50 OR S51 OR S52 OR S53 OR S54 OR S55 OR S56 OR S57 OR S58 OR S59 OR S60 OR S61
S61	"help#line"
S60	"hotline*" OR (MH "Telephone Information Services")
S59	"call cent*"
S58	"consensus development conference"
S57	"congress*"
S56	"survivor*"
S55	(MH "Support Groups") OR "support groups"
S54	"psychosocial support system*"
S53	"social support"
S52	(MH "Community Networks") OR "community network*"
S51	"internet forum*"
S50	"doctor patient communication*"
S49	"breaking bad news"
S48	(MH "Researcher-Subject Relations")
S47	(MH "Dentist-Patient Relations") OR (MH "Physician-Patient Relations") OR (MH "Nurse-Patient Relations")
S46	(MH "Education, Nursing") OR (MH "Education, Nursing, Continuing") OR (MH "Education, Nursing, Graduate") OR (MH "Education, Nursing, Associate") OR (MH "Education, Nursing, Diploma Programs") OR (MH "Education, Nursing, Baccalaureate")

S45	"dental education" OR (MH "Education, Dental") OR (MH "Dental Health Education")
S44	(MH "Caregivers") OR "caregiver*"
S43	(MH "Education, Medical")
S42	"podcast*"
S41	"webcast*" OR (MH "Webcasts")
S40	"handout*"
S39	"brochure*"
S38	"flyer*"
S37	"pamphlet" OR (MH "Pamphlets")
S36	"leaflet*"
S35	"snapchat*"
S34	"instagram"
S33	(MH "Social Networks") OR (MH "Social Networking") OR (MH "Social Network Analysis (Saba CCC)") OR "social network*"
S32	"wordpress"
S31	"facebook"
S30	"tweet*"
S29	"twitter"
S28	(MH "Internet") OR "internet"
S27	"blogging" OR (MH "Blogs")
S26	"media campaign*" OR (MH "Social Media")
S25	"cd*" OR (MH "CD ROM")
S24	(MH "Videodiscs") OR (MH "Audiorecording") OR (MH "Videorecording") OR "videodisc recording*"
S23	(MH "Television") OR "television"

S22	(MH "Radio") OR "radio"
S21	(MH "Motion Pictures") OR "motion picture*"
S20	"mass media" OR (MH "Communications Media")
S19	(MH "Patient Education")
S18	(MH "Health Promotion") OR "health promotion*"
S17	(MH "Health Fairs") OR "health fair*"
S16	(MH "Consumer Health Information") OR "consumer health information"
S15	(MH "Health Education") OR "health education"
S14	(MH "Medical Illustration") OR "medical illustration"
S13	(MH "Writing") OR (MH "Medical Writing") OR (MH "Report Writing")
S12	"literacy"
S11	"information seeking behavio#r"
S10	"cell phone use"
S9	"communication"
S8	S1 OR S2 OR S3 OR S4 OR S5 OR S6 OR S7
S7	"rare carcinoma*"
S6	"rare neoplasm*"
S5	"rare sarcoma*"
S4	"rare adenocarcinoma*"
S3	"rare tumour*"
S2	"rare tumor*"
S1	"rare cancer*"

PubMed Search

(communication*) AND ((((((("rare cancer*") OR "rare carcinoma*") OR "rare neoplas*") OR "rare malignan*") OR "rare adenocarcinoma*") OR "rare sarcoma*") OR "rare tumor*") OR "rare tumour*")
